# Evolution in optical molecular imaging techniques guided nerve imaging from 2009 to 2023: a bibliometric and visualization analysis

**DOI:** 10.3389/fneur.2024.1474353

**Published:** 2025-01-22

**Authors:** Wenkai Liang, Yan Liu, Erlong Jia, Xiaofeng Yang, Shufeng Han, Jinzheng Wei, Wei Zhao

**Affiliations:** ^1^Department of Orthopedics, First Hospital of Shanxi Medical University, Taiyuan, Shanxi, China; ^2^Department of Critical Care Medicine, First Hospital of Shanxi Medical University, Taiyuan, Shanxi, China; ^3^Department of Urology, First Hospital of Shanxi Medical University, Taiyuan, Shanxi, China; ^4^School of Basic Medicine, Qingdao Medical College of Qingdao University, Qingdao, China

**Keywords:** optical molecular imaging techniques, nerve imaging, fluorescence guided surgery, bibliometric, web of science

## Abstract

**Background:**

Recent years, the use of optical molecular imaging (OMI) techniques guided nerve imaging has made significant progress. However, a comprehensive bibliometric analysis in this field is currently lacking. In this study, we aim to shed light on the current status, identify the emerging hot topics, and provide valuable insights for researchers within this field.

**Methods:**

In this study, we collected 414 research via the Web of Science Core Collection (WoSCC) from 2009 to 2023. CiteSpace, VOSviewer and R package “bibliometrix” were used for analysis of countries, institutions, journals, etc., to evaluate the trends.

**Results:**

The amounts of publications in relation to OMI guided nerve imaging has been increasing. United States and China contributed to over 60% of the publications. The Shanghai Jiao Tong University contributed the highest number of publications. Investigative Ophthalmology and Visual Science is considered the most prestigious and prolific journal in the field. It is also widely regarded as the most cited journal. Among the top 10 authors in terms of output, Hehir CAT has the highest number of citations. The “neurosciences neurology,” “science technology other topics,” and “ophthalmology” are representative research areas. The main cluster of keywords in this field includes “axonal regeneration,” “mouse,” and “optical coherence tomography.”

**Conclusion:**

This bibliometric investigation offers a comprehensive portrayal of the structure of knowledge and the progression patterns, presents an all-encompassing synthesis of findings, discerns and illustrates the forefront within OMI guided nerve imaging for the first time. It will provide a valuable reference for relevant scholars.

## 1 Introduction

Iatrogenic nerve injury is a common and serious complication in surgical procedures (1, 2). This is typically caused by the surgeon’s inability to clearly identify nerve tissue, resulting in irreversible damage due to inappropriate manipulation (3). Nerve injuries resulting from surgery can have devastating effects on patients’ sensation, movement, and other bodily functions, thus negatively impacting their quality of life (4). Therefore, the identification and preservation of nerve tissue are crucial considerations in many surgical procedures. Currently, due to the clinical lack of direct imaging methods for peripheral nerves, the recognition and functional monitoring of peripheral nerves mainly rely on clinical history, physical examination, electromyography, magnetic resonance imaging (MRI), computed tomography (CT), and high-resolution ultrasound (5, 6). However, these approaches frequently exhibit restricted sensitivity and specificity, and a few might entail potential radiological hazards, making it challenging to achieve real-time precise identification and localization of nerve tissue during surgery.

In 2009, Professor Roger YT firstly proposed the conception of OMI guided surgery at the World Molecular Imaging Congress (7). This marked the realization of intraoperative real-time identification and localization of target areas using fluorescent dyes. After more than a decade of development, this technique has become an attractive tool in surgical procedures (8). By utilizing fluorescent dyes, this technique assists surgeons in achieving real-time fluorescence imaging of target tissues for surgical decision-making. Compared to the aforementioned MRI, CT, and high-resolution ultrasound methods, this technique offers high sensitivity and spatial resolution, generating highly intuitive fluorescence images that can help reduce surgical training costs and operating time. Additionally, the imaging equipment is simple in structure and easy to use (9). To date, a considerable number of related imaging devices have been applied in endoscopic, laparoscopic, and open surgeries. Fluorescein sodium, methylene blue, indocyanine green (ICG), and other fluorescent dyes have been successfully applied in tumor labeling (10), vascular imaging (11), ureteral imaging (12), sentinel lymph node identification (9, 13), and bile duct imaging (14). However, the bundled structure of nerve fibers presents a difficulty in staining them with fluorescent probes when compared to tubular structures such as blood vessels, ureters, and bile ducts. This has stimulated research interest in OMI guided nerve imaging. Nevertheless, the global literature on this topic remains limited, making it necessary to evaluate and summarize its current status and trends.

In recent years, bibliometric analysis has effectively been employed to examine extensive quantities of scientific research data, ascertain emerging patterns of development (15). Significantly, this approach enables the synthesis of the evolutionary trajectory of publications, anticipation of research focal points, and subsequent evaluation of the cutting-edge advancements in particular domains by employing citation networks (16, 17). Upon investigation, no similar analytical reports regarding OMI guided nerve imaging were found. Therefore, we utilized bibliometric analysis to fill the lacking in this field. We comprehensively analyzed relevant literature from 2009 to 2023 and performed visual analysis to identify significant characteristics and predict future hotspots.

## 2 Materials and methods

### 2.1 Data sources and search approaches

The WoSCC database is regarded as one of the most comprehensive and authoritative platforms, comprising more than 12,000 academic journals from around the world (18). In this study, we analyzed data via bibliometric methods based on previous research (19–21). All literature used in this study was extracted from the WoSCC. The search period was set from January 1, 2009, to December 31, 2023, with the following search formula: TS = ((AB = (fluorescence imaging)) OR AB = (optical molecular imaging)) AND AB = (nerve imaging).

**Figure 1:**
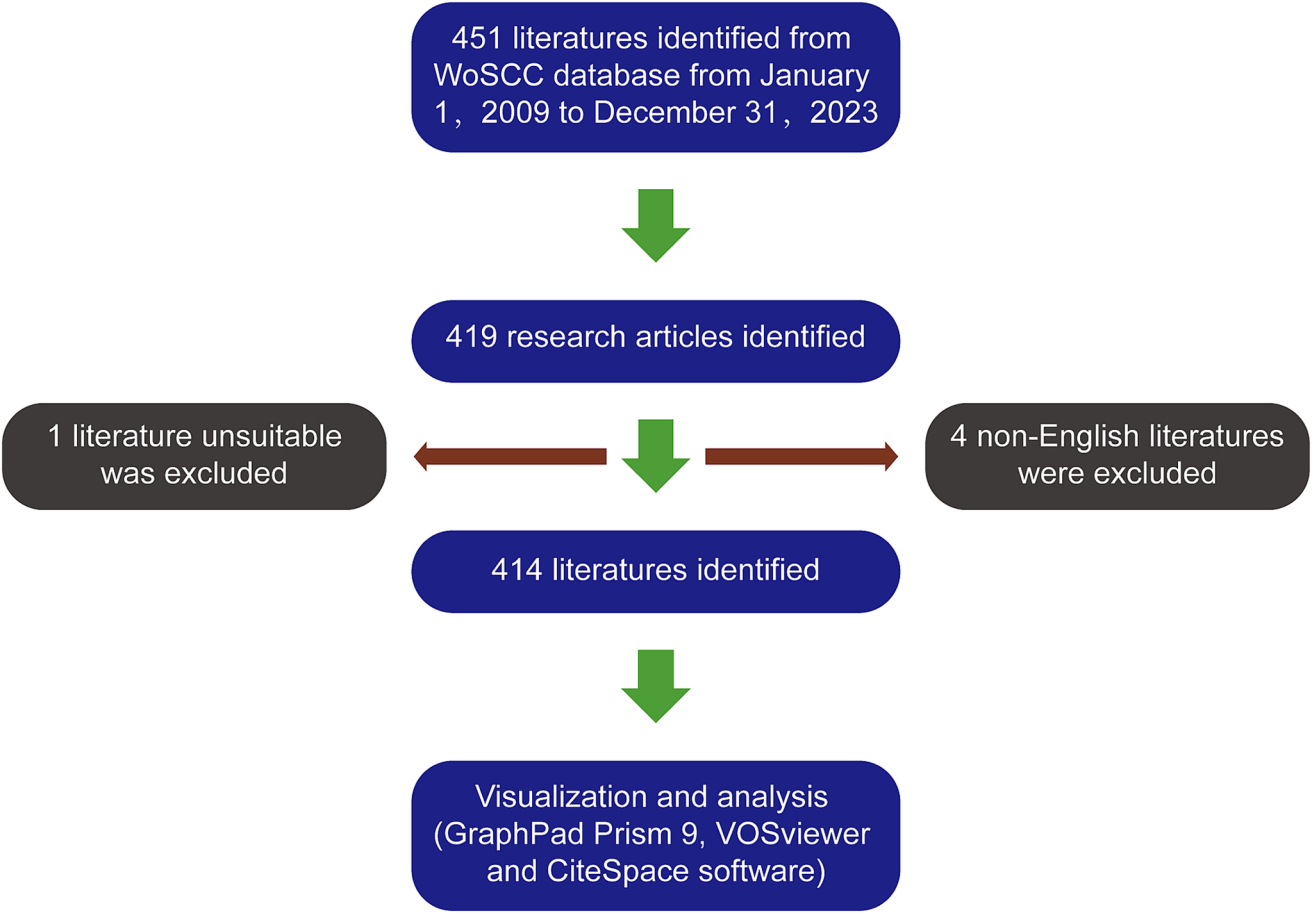
The article selection process.

We will consider the following criteria for inclusion: (1) research publications that specifically examine the utilization of OMI techniques guiding nerve imaging; (2) research articles as the document type; (3) the studies must be written in English. The following criteria will be excluded: (1) publications that have no relevance to OMI techniques guiding nerve imaging; (2) reviews, conference abstracts, proceedings, book chapters, editorials, letters, news articles, and similar content (Figure 1). The database obtained after filtering is detailed in Supplementary File 1.

During the process of conducting research, relevant details pertaining to particular nations in the WoSCC repository were refined by indexing them. All relevant data from the literature, including publication year, title, author, nationalities, affiliations, abstracts, keywords, and journal names, were saved from the WoSCC database. Both Wenkai Liang and Jinzheng Wei conducted separate searches and retrieved all the required information. The final decision is made by consulting the corresponding authors with discrepancies. GraphPad Prism 9 software was used for data organization and analysis.

### 2.2 Bibliometric analysis and visualization

We utilized VOSviewer 1.6.14 software to construct and visualize collaboration analysis and network analysis graphs in our study. In above graphs, every node means a project (like co-cited references or keywords), and the size of the node means the amounts of publications. The color of the nodes represents various years. The thickness of the link between nodes indicates the strength of the collaborative or co-citation relationships. Additionally, CiteSpace 6.1.2 was employed for constructing dual-map overlay of journals, cluster analysis, and detecting references and keywords with strong citation bursts. We choose the R software package “bibliometrix” to draw the geographical network map, Bradford’s Law Core Sources map, the Lotka’s Law distribution map, the Authors’ production over are drawn the time map and the Three-field plot of the keywords analysis.

## 3 Results

### 3.1 Global contributions to the field

**Figure 2:**
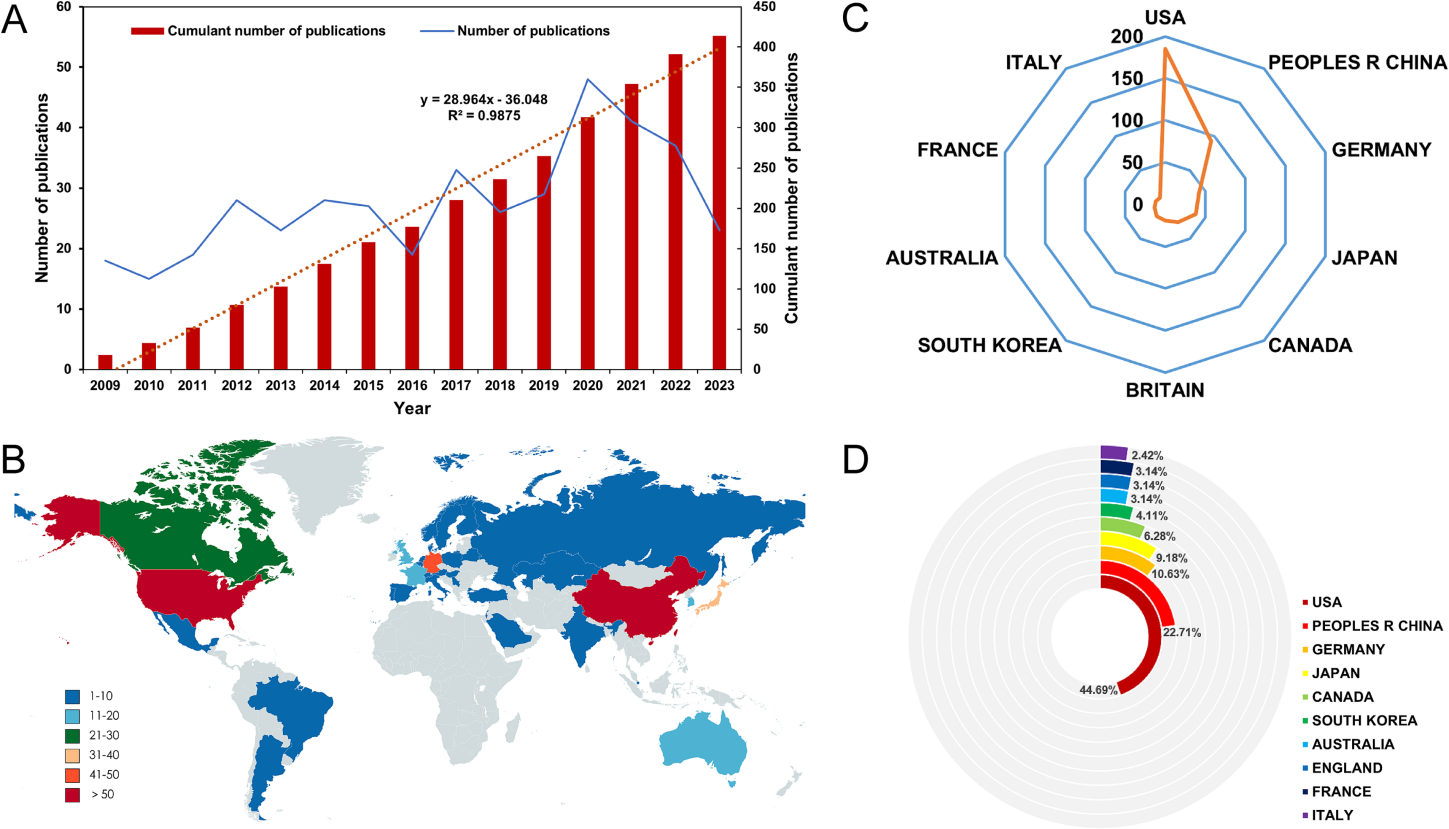
**(A)** The model fitting curves in cumulant number of publications in the field. **(B)** World map showing the distribution of publications. **(C)** Radar chart of the top 10 countries in terms of the number of publications in the field. **(D)** Pie chart of the top 10 countries in terms of the number of publications in the field by R software.

According to our search approach, 451 articles were collected from 2009 to 2023. Among them, there were 419 research articles. After excluding 4 non-English articles and 1 article unrelated to the topic, we identified a total of 414 articles (Figure 1). The global literature in this field has shown a linear growth trend, increasing from 18 articles in 2003 to 414 articles in 2023. To predict future trends, a logistic regression model was created to a model fitting curves in cumulant number of publications. Based on this curve, the annual growth trend fits the fitted curve *y* = 28.964*x*–336.048 (*R*^2^ = 0.9875) (Figure 2A).

### 3.2 Analysis of countries and institutions contributing to the field

**Figure 3:**
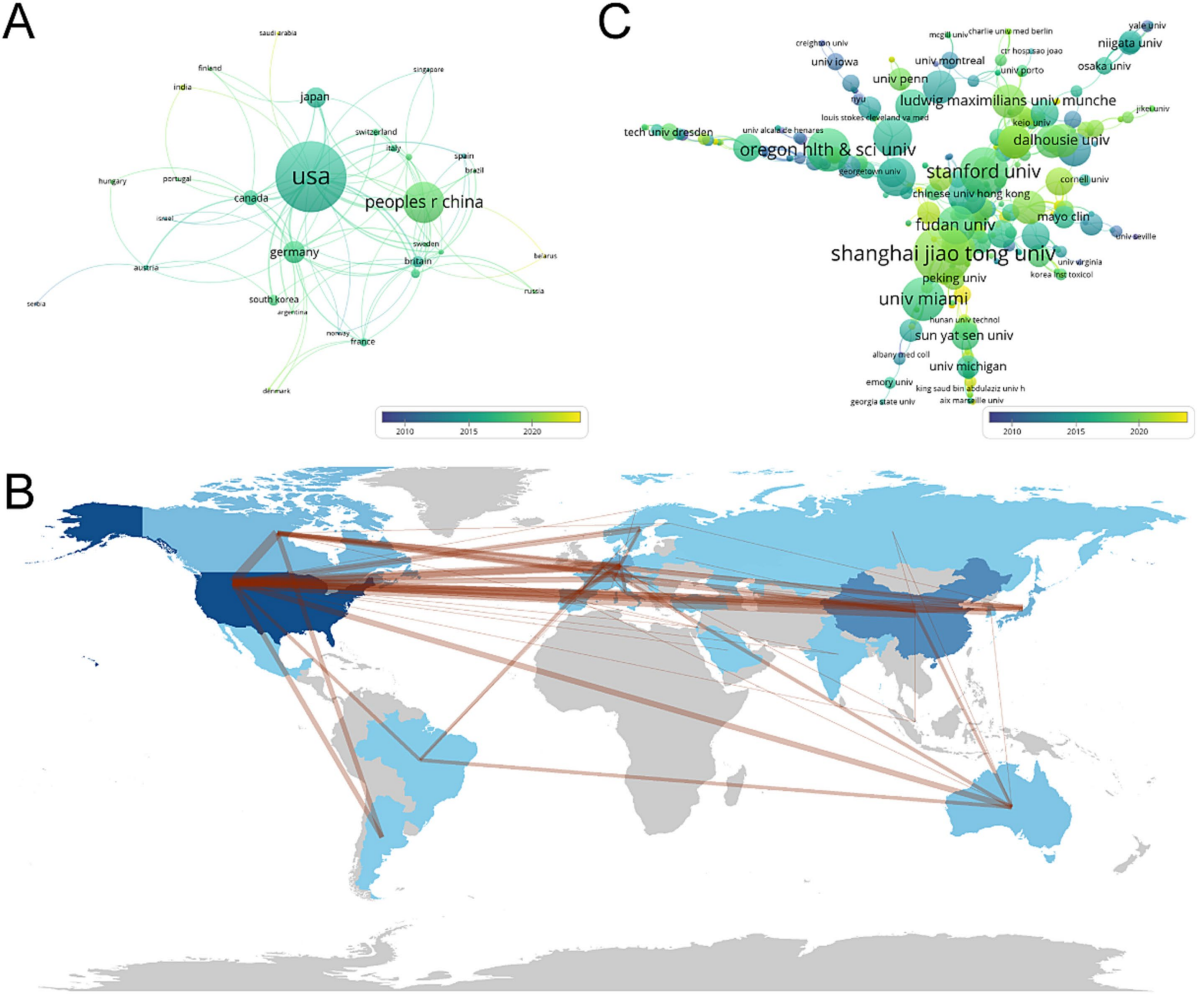
**(A)** Chart for analysis of country cooperation by VOSviewer. **(B)** The geographical network map by VOSviewer. **(C)** Chart for analysis of institutional cooperation by R software. Nodes represent countries or institutions, connected by lines that represent collaboration. The number of published articles is represented by the size of the node. The lines between nodes represent cooperative relationships, while the thickness of the connecting lines represents the strength of their cooperation. From 2009 to 2023, the color changed from purple to yellow.

A total of 35 countries have made contributions to the literature in this field (Figure 2B). The USA has the highest number of published papers, with 185 records (44.69%), followed by China (93 records, 22.46%), Germany (42 records, 10.15%), Japan (38 records, 9.18%), and Canada (26 records, 6.8%) (Figure 2C and Supplementary Table S1). The top 10 countries in terms of publication volume are distributed across North America, Western Europe, and Asia (Figure 2D and Supplementary Table S1). Among them, the USA and China account for more than 60% of the publication counts, far surpassing other countries. As shown in Supplementary Table S1, the USA has the highest total citation count with 4,828 citations, followed by China with 1,590 citations. Although the contribution of the Britain to the total publication count is less than 5%, it has the highest average citation (40.21). The connections between nodes depict the collaboration map of countries involved in this particular field. Node size signifies the overall publication count (Figure 3A). It is worth noting that the USA and China are also the top two countries involved in collaborations with other countries (Figure 3B).

A total of 633 institutions globally have contributed to the literature in this field. As shown in Supplementary Table S2, the top 10 institutions in terms of productivity are all from China, the USA, and Canada. Shanghai Jiao Tong University has the highest contribution with 11 articles and 102 citations, followed by Stanford University with 9 articles and 116 citations. Among the top 10 research institutions in terms of productivity, Oregon Health and Science University has the highest average citation counts (32.75), followed by the University of Toronto (29.71) and the University of Miami (25.75). Additionally, the institutional collaboration analysis shows that Shanghai Jiao Tong University, Stanford University, and Oregon Health and Science University are the top three institutions involved in collaborations with other institutions (Figure 3C).

### 3.3 Analysis of journals and research areas

**Figure 4:**
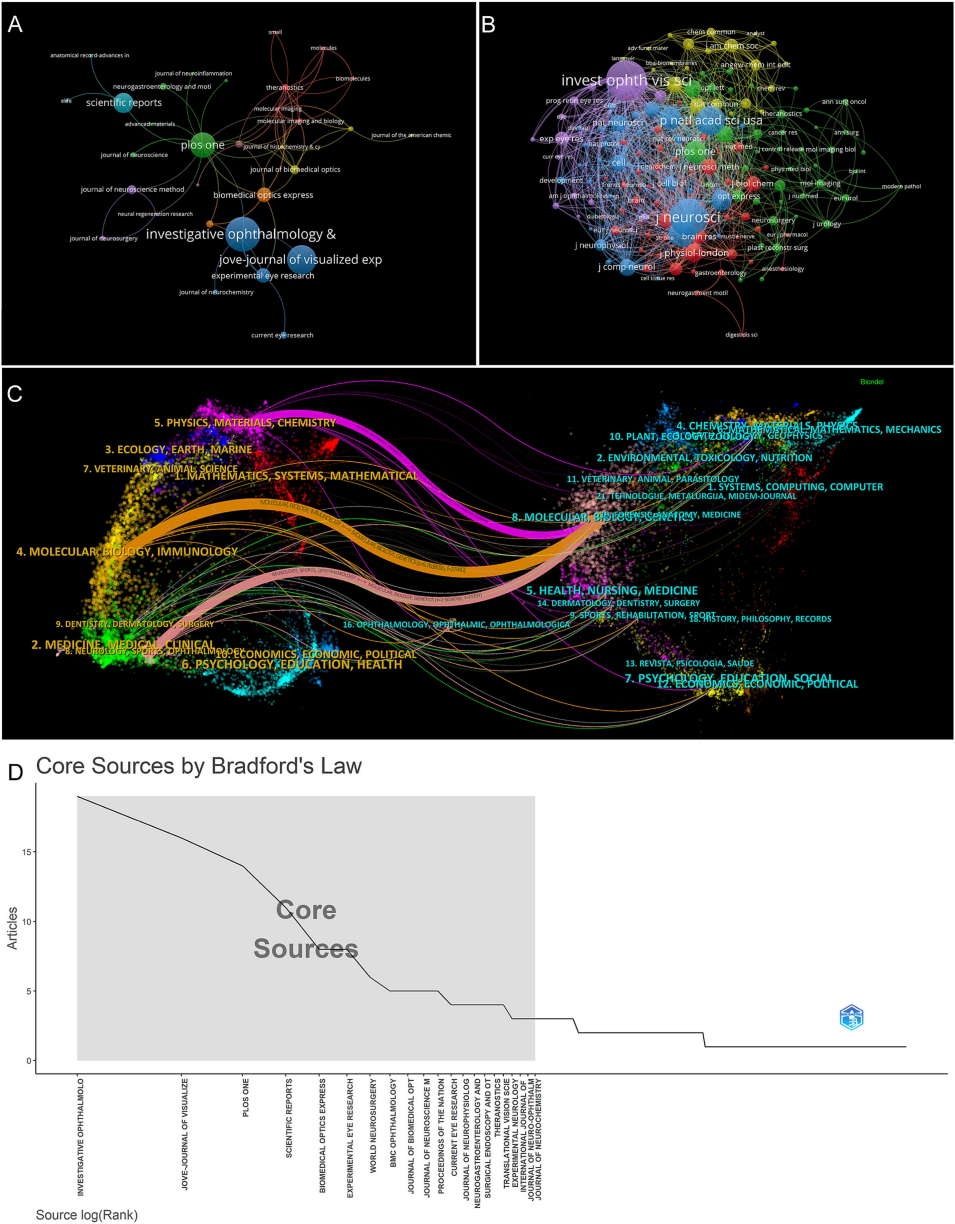
**(A)** Network analysis graph of journals cited more than 2 times by VOSviewer. **(B)** Network analysis graph of journals co-cited more than 20 times by VOSviewer. **(C)** A dual-map overlay of journals depicting citation relationships between cited and citing journals based on CiteSpace. **(D)** Bradford’s Law Core Sources by R software. The shaded sections represent journals that account for the top third of the total number of publications.

The research field has seen publications in a total of 248 journals from 2009 to 2023. Table 1 displays the top 10 journals with the highest number of published articles and their latest Impact Factors (IF). Investigative Ophthalmology and Visual Science has the highest number of publications (19 articles, accounting for 4.59% of all papers), followed by Jove-Journal of Visualized Experiments (16 records, 3.86%), PLoS One (14 records, 3.38%), Scientific Reports (11 records, 2.66%), and Biomedical Optics Express (8 records, 1.93%). Among the top 10 journals, Scientific Reports has the highest IF (4.6), followed by Investigative Ophthalmology and Visual Science (4.4), and PLoS One (3.7). There are a total of 64 journals with more than 2 citations, and the top 5 journals with the highest total link strength are Molecular Imaging, PLoS One, Theranostics, Biomedical Optics Express, and Experimental Eye Research (Figure 4A). The cumulative number of articles in the top 21 journals represents one-third of the total number of publications (Figure 4D).

**Table 1:** Top 10 journals that contributed publications.

Rank	Journal	Record counts	Percentage (%, N/414)	Total citations	Average citation	IF
1	Investigative Ophthalmology and Visual Science	19	4.59%	463	24.37	4.4
2	Jove-Journal of Visualized Experiments	16	3.86%	130	8.13	1.2
3	PLoS One	14	3.38%	336	24.00	3.7
4	Scientific Reports	11	2.66%	398	36.00	4.6
5	Biomedical Optics Express	8	1.93%	202	25.25	3.4
6	Experimental Eye Research	8	1.93%	161	20.13	3.4
7	World Neurosurgery	6	1.45%	218	36.33	2
8	BMC Ophthalmology	5	1.21%	76	15.20	2
9	Journal of Biomedical Optics	5	1.21%	23	4.60	3.5
10	Journal of Neuroscience Methods	5	1.21%	215	43.00	3

The field is covered by a total of 163 journals, with 20 or more citations each (Figure 4B). Table 2 displays the top 10 ranked journals, including Investigative Ophthalmology and Visual Science (536), Journal of Neuroscience (486), PNAS (335), Neuron (302), and Nature (228). Journal of Neuroscience, PNAS, Neuron, Nature, Science, and Journal of Physiology, which are highly cited journals, did not make it into the top 10 list in terms of output.

**Table 2:** Top 10 cited journals.

Rank	Journal	Total citations	IF
1	Investigative Ophthalmology and Visual Science	536	4.4
2	Journal of Neuroscience	486	5.3
3	PNAS	335	11.1
4	Neuron	302	16.2
5	Nature	228	64.8
6	PLoS One	227	3.7
7	Science	204	56.9
8	Journal of Physiology	169	5.5
9	Journal of Biomedical Optics	154	3.5
10	Biomedical Optics Express	152	3.4

The included publications are classified into 57 research fields. Neurosciences neurology (88 records, 21.26%), science technology other topics (70 records, 16.91%), and ophthalmology (61 records, 14.73%) represent the top three research fields (Table 3). The citation relationship between citing and cited journals is depicted using a dual-map overlay of journals (Figure 4C), as shown in previous studies (22). The left cluster (research front) represents the publishing area of the citing journals, and the right cluster represents the publishing area of the cited journals. The spline waves connect them in a left-to-right manner, displaying citation relationships through colored paths. The primary citation paths are depicted by a single purple line, a single orange line, and a single pink line. The purple path indicates papers published in physics, materials, and chemistry, primarily cited by the molecular, biology, and genetics fields. The orange path indicates papers published in molecular, biology, and immunology fields, primarily cited by the molecular, biology, and genetics fields. The pink path indicates papers published in neurology, sports, and ophthalmology fields, primarily cited by the molecular, biology, and genetics fields.

**Table 3:** Top 10 well-represented research areas.

Rank	Research areas	Record counts	Percentage (%, N/414)	Total citations
1	Neurosciences Neurology	88	21.26%	2,053
2	Science Technology Other Topics	70	16.91%	1,588
3	Ophthalmology	61	14.73%	1,241
4	Biochemistry Molecular Biology	50	12.08%	1,357
5	Chemistry	42	10.14%	904
6	Surgery	34	8.21%	517
7	Optics	26	6.28%	429
8	Radiology Nuclear Medicine Medical Imaging	24	5.80%	467
9	Materials Science	23	5.56%	660
10	Cell Biology	19	4.59%	341

### 3.4 Analysis of author and funding source

**Figure 5:**
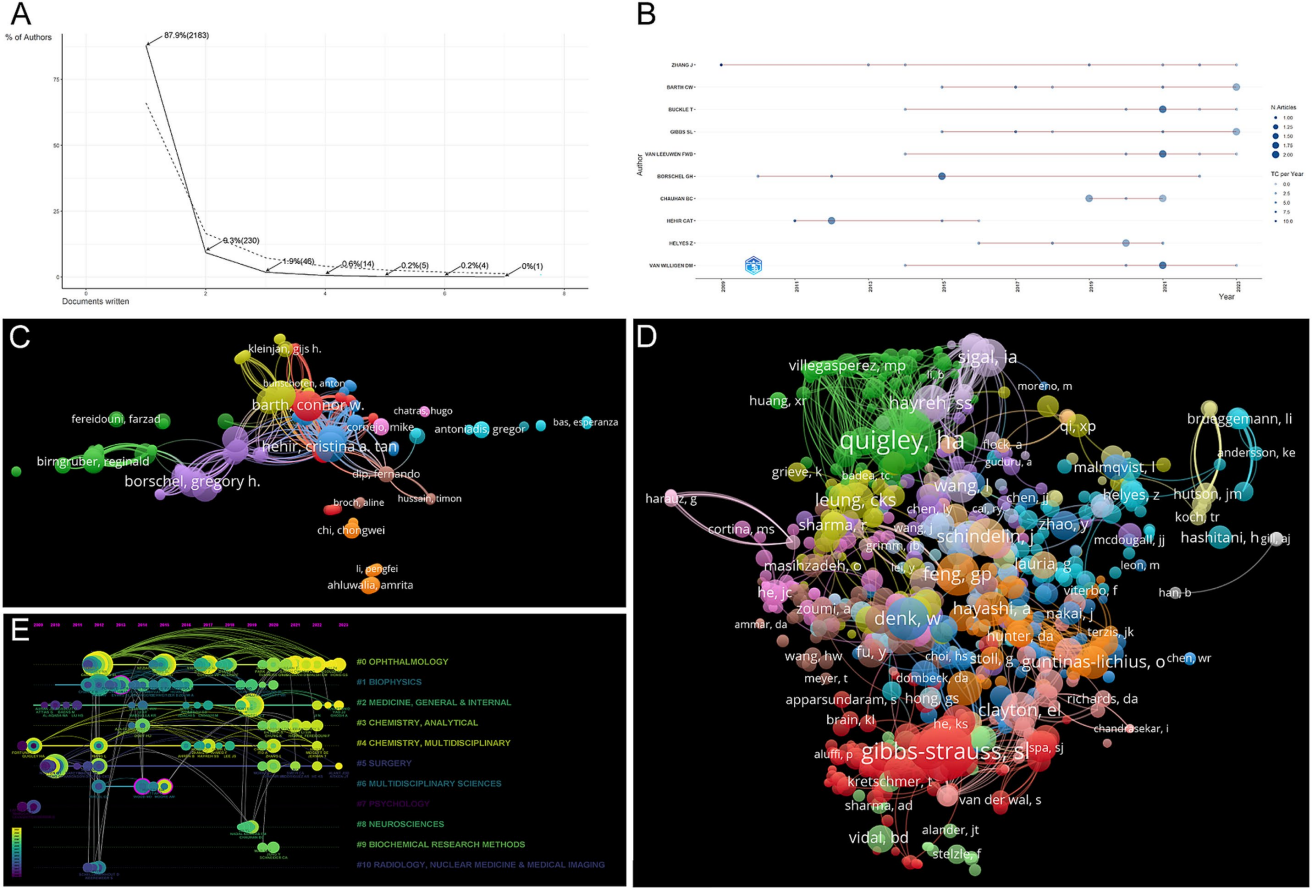
**(A)** The Lotka’s Law distribution map by R software. **(B)** Authors’ production over the time by R software. **(C)** Collaboration analysis graph author by VOSviewer. **(D)** Network analysis graph of co-cited authors of publications by VOSviewer. **(E)** Author timeline visualization from 2009 to 2023. Nodes indicate co-authors or co-cited authors based on CiteSpace. The lines connecting nodes represent the co-citation relationship. The node area increases with the increase of co-citation times.

At the same time, we drew the Lotka’s Law distribution map. The ordinate indicates the proportion of authors of different literatures to all authors, and the abscissa indicates the number of documents (Figure 5A). The dotted line in the graph represents the ideal image of Lotka’s Law. From the figure, we can see that there are 2,183 scholars who have published only 1 paper, accounting for 87.9% of the total. The number of scholars who published 2 papers is 230, accounting for 9.3% of the total. The number of authors and literature in this field is similar to the dotted line in the graph, generally conforming to the general laws of Lotka’s Law. This indicates that most scholars in this field are still at the initial stage of research and their studies are not yet deep.

Figure 5B and Supplementary Table S3 show the top 10 authors with the highest publications over that time period. Zhang J has published the most papers in the field for many years, with his first article published in 2009 and his last in 2023. However, Hehir CAT, Borschel G, and Cotero VE are currently the three most cited in the field. Table 4 presents the top 10 authors in terms of citation count in the field. The 10 authors are from the USA, Canada, Hungary, and the Netherlands. Hehir CAT (184), Borschel G (158), and Cotero VE (109) rank as the top three in terms of total citations. Interestingly, none of the authors in the aforementioned rankings are from China. It also shows that the quality of articles published by Chinese scholars in this field needs to be improved.

**Table 4:** Top 10 authors with the most citations.

Rank	Author	Country	Total citations
1	Hehir CAT	USA	184
2	Borschel G	CANADA	158
3	Cotero VE	USA	109
4	Yazdanfar S	USA	90
5	Gibbs SL	USA	88
6	Bajaj A Barth C	USA	86
7	Barth C	USA	85
8	Wood MD	CANADA	74
9	Helyes Z	HUNGARY	53
10	Van Leeuwen FWB	NETHERLANDS	50

A network analysis graph depicting the cooperative analysis of authors was conducted, showcasing the co-cited author relationships (Figures 5C,D). The most prominent co-cited authors in the field are Gibbs SL (45), Quigley HA (38), Whitney MA (19), Feng GP (19), and Denk W (19). Notably, Gibbs SL appears in both Supplementary Table S3 and Table 4.

In addition, the analysis of co-citations over time is presented in the timeline visualization (Figure 5E). Early research hotspots in the field included “medicine, general and internal” (cluster 2), “chemistry, multidisciplinary” (Cluster 4), “surgery” (Cluster 5), and “psychology” (Cluster 7). Recent and mid-term research hotspots encompassed “ophthalmology” (cluster 0), “biophysics” (cluster 1), “medicine, general and internal” (Cluster 2), “chemistry, analytical” (Cluster 3), “chemistry, multidisciplinary” (Cluster 4), “surgery” (Cluster 5), “multidisciplinary sciences” (Cluster 6), “neuroscience” (Cluster 8), “biochemical research methods” (Cluster 9), and “radiology, nuclear medicine and medical imaging” (Cluster 10). Current research focuses on “ophthalmology” (Cluster 0), “medicine, general and internal” (Cluster 2), and “surgery” (cluster 5), with some terms overlapping with the early research hotspots (Clusters 2 and 5), indicating sustained research interests over the past decades.

Furthermore, an analysis regarding the funding sources for publications within this particular field was carried out. A detailed presentation of the top 10 sources can be found in Table 5. Out of the total publications, 117 (28.26%) were funded by the USA Department of Health Human Services, followed by the National Institutes of Health with 116 publications (28.02%), and the National Natural Science Foundation of China with 49 publications (11.84%).

**Table 5:** Top 10 funding sources for publications.

Rank	Funds	Record Counts	Percentage (%, N/414)	Country
1	United States Department of Health Human Services	117	28.26	USA
2	National Institutes of Health	116	28.02	USA
3	National Natural Science Foundation of China	49	11.84	CHINA
4	Research to Prevent Blindness	21	5.07	USA
5	Japan Society for The Promotion of Science	19	4.59	JAPAN
6	Ministry of Education Culture Sports Science and Technology	19	4.59	JAPAN
7	Grants in Aid for Scientific Research	17	4.11	JAPAN
8	Canadian Institutes of Health Research	15	3.62	CANADA
9	German Research Foundation	14	3.38	GERMANY
10	National Science Foundation	14	3.38	USA

### 3.5 Analysis of citation and co-citation

**Figure 6:**
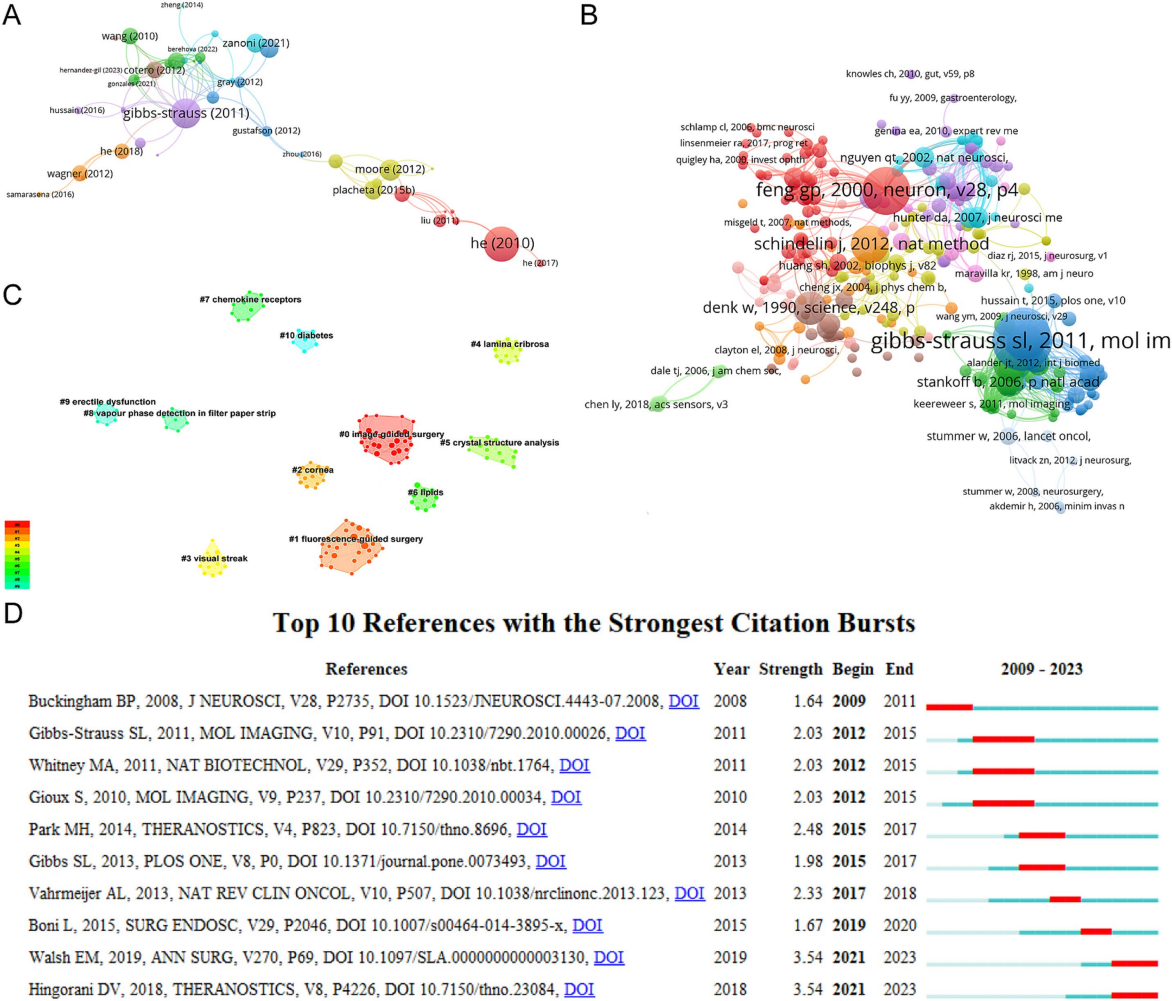
**(A)** Network citation analysis graph more than 1 time by VOSviewer. **(B)** Network co-citation analysis more than 3 times by VOSviewer. **(C)** Clustering analysis of the co-citation network based on CiteSpace. **(D)** Top 10 references with the strongest citation bursts for publications based on CiteSpace.

We conducted a network citation analysis using VOSviewer on a total of 369 references with more than 1 time in the field (Figure 6A). The top 10 articles with the highest number of citations are presented in Table 6. There were 167 citations for “Cytotoxicity of oxycodone and morphine in human neuroblastoma and mouse motoneuronal cells: a comparative approach” (DOI: 10.1213/ane.0b013e31819385e1), followed by “Rapid transport within cerebral perivascular spaces underlies widespread tracer distribution in the brain after intranasal administration” (DOI: 10.1038/jcbfm.2014.215), with 165 citations, and “*In vivo* imaging reveals a phase-specific role of STAT3 during central and peripheral nervous system axon regeneration” (DOI: 10.1073/pnas.1015239108), with 161 citations.

**Table 6:** Top 10 research articles with the most citations.

Rank	DOI	Journal	IF	Total citations
1	10.1038/jcbfm.2014.215	JCBFM	6.3	165
2	10.1073/pnas.1015239108	PNAS	11.1	161
3	10.1073/pnas.1711734114	PNAS	11.1	132
4	10.1016/j.visres.2009.01.010	Vision Research	1.8	129
5	10.1016/j.jneumeth.2010.12.011	Journal of Neuroscience Methods	3	126
6	10.1039/c6nr07581a	Nanoscale	6.7	121
7	10.1016/j.exer.2010.07.007	Experimental Eye Research	3.4	119
8	10.1038/nn.3067	Nature Neuroscience	25	113
9	10.1017/S1431927614001329	Microscopy and Microanalysis	2.8	112
10	10.1016/j.expneurol.2013.03.001	Experimental Neurology	5.3	106

We visualized the co-citation references using VOSviewer (Figure 6B). Table 7 shows the top 10 references with the highest number of co-citations. References published by the corresponding authors Frangioni JV (23), Nguyen QT (19), Nguyen QT (18), Hollister SJ (144), and Hutmacher DW (139) rank among the top three. Next, we used CiteSpace to cluster the co-citation reference based on indexing terms, resulting in 11 main clusters: “image-guided surgery,” “fluorescence-guided surgery,” “cornea, “lamina cribrosa,” “crystal structure analysis,” “lipids,” “chemokine receptors,” “vapor phase detection in filter paper strip,” “erectile dysfunction,” and “diabetes” (Figure 6C).

**Table 7:** Research articles with the co-citations more than 8.

Rank	DOI	Corresponding author	Journal	IF	Publication Year	Total co-citations
1	10.2310/7290.2010.00026	Frangioni JV	Molecular Imaging	2.8	2011	23
2	10.1038/nbt.1764	Nguyen QT	Nature Biotechnology	46.9	2012	19
3	10.1016/s0896-6273(00)00084-2	Nguyen QT	Neuron	16.2	2000	18
4	10.7150/thno.8696	Choi HS	Theranostics	12.4	2014	14
5	10.1038/nmeth.2019	Cardona A	Nature Methods	48	2012	14
6	10.1007/s11307-012-0555-1	Hehir CAT	Molecular Imaging and Biology	3.1	2012	12
7	10.1126/science.2321027	Webb WW	Science	56.9	1990	12
8	10.1073/pnas.0600769103	Stankoff B	PNAS	11.1	2006	10

References with citation bursts are considered valuable indicators of frequently cited literature within a specific field during a certain period of time (23). The top 10 references with the strongest citation bursts, along with their citation durations, are displayed in Figure 6D. The article titled “Fluorescence imaging of nerves during surgery” published in 2019 and the article titled “Nerve-targeted probes for fluorescence-guided intraoperative imaging” published in 2018 ranked first in terms of citation bursts (strength = 3.54). Additionally, the citation bursts of the articles titled “Nerve-highlighting fluorescent contrast agents for image-guided surgery” and “Image-guided surgery using invisible near-infrared light: fundamentals of clinical translation” had the longest duration, spanning from 2012 to 2015.

### 3.6 Co-occurrence analysis of keywords

**Figure 7:**
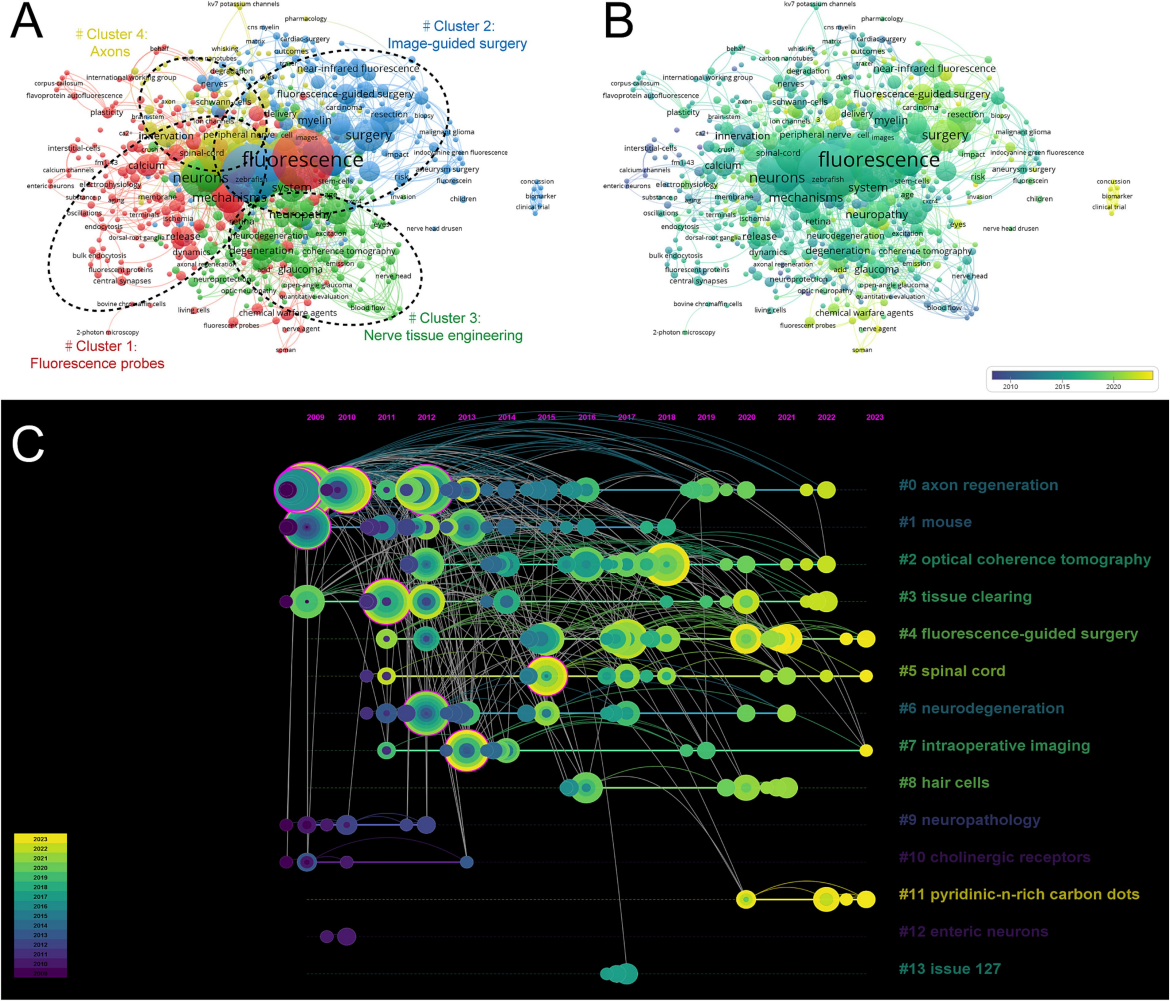
**(A)** Network analysis graph of keywords by VOSviewer. **(B)** Distribution of keywords according to average publication year by VOSviewer. **(C)** Keyword timeline visualization from 2009 to 2023 by CiteSpace.

Using VOSviewer, we conducted a comprehensive co-occurrence analysis of keywords to effectively identify the current research frontiers within the field. Our analysis led us to categorize these frontiers into four distinct clusters: “Fluorescence” (Cluster 1), “Image-guided surgery” (Cluster 2), “Nerve tissue engineering” (Cluster 3), and “Axons” (Cluster 4) (Figure 7A). A network analysis graph was generated using VOSviewer, with colors indicating the average publication year (dark blue: earlier; yellow: later) (Figure 7B). A total of 651 keywords were obtained, with a minimum occurrence threshold set at 2 times. The top 5 most frequently occurring keywords were “fluorescence” (Total link strength: 121), “in-vivo” (Total link strength: 114), “expression” (Total link strength: 72), “neurons” (Total link strength: 56), and “microscopy” (Total link strength: 51).

We got the keyword timeline visualization from 2009 to 2023 by CiteSpace (Figure 7C). Fourteen main clusters were identified: “axonal regeneration” (Cluster 0), “mouse” (Cluster 1), “optical coherence tomography” (Cluster 2), “tissue clearing” (Cluster 3), “fluorescence-guided surgery” (Cluster 4), “spinal cord” (Cluster 5), “neurodegeneration” (Cluster 6), “intraoperative imaging” (Cluster 7), “hair cells” (Cluster 8), “neuropathology” (Cluster 9), “cholinergic receptors” (Cluster 10), “pyridinic-n-rich-carbon dots” (Cluster 11), “enteric neurons” (Cluster 12), and “issue” (Cluster 13). Clusters 0, 1, 3, 9, 11, and 12 represent previous research hotspots, while clusters 4, 5, 7, and 11 represent current research hotspots.

**Figure 8:**
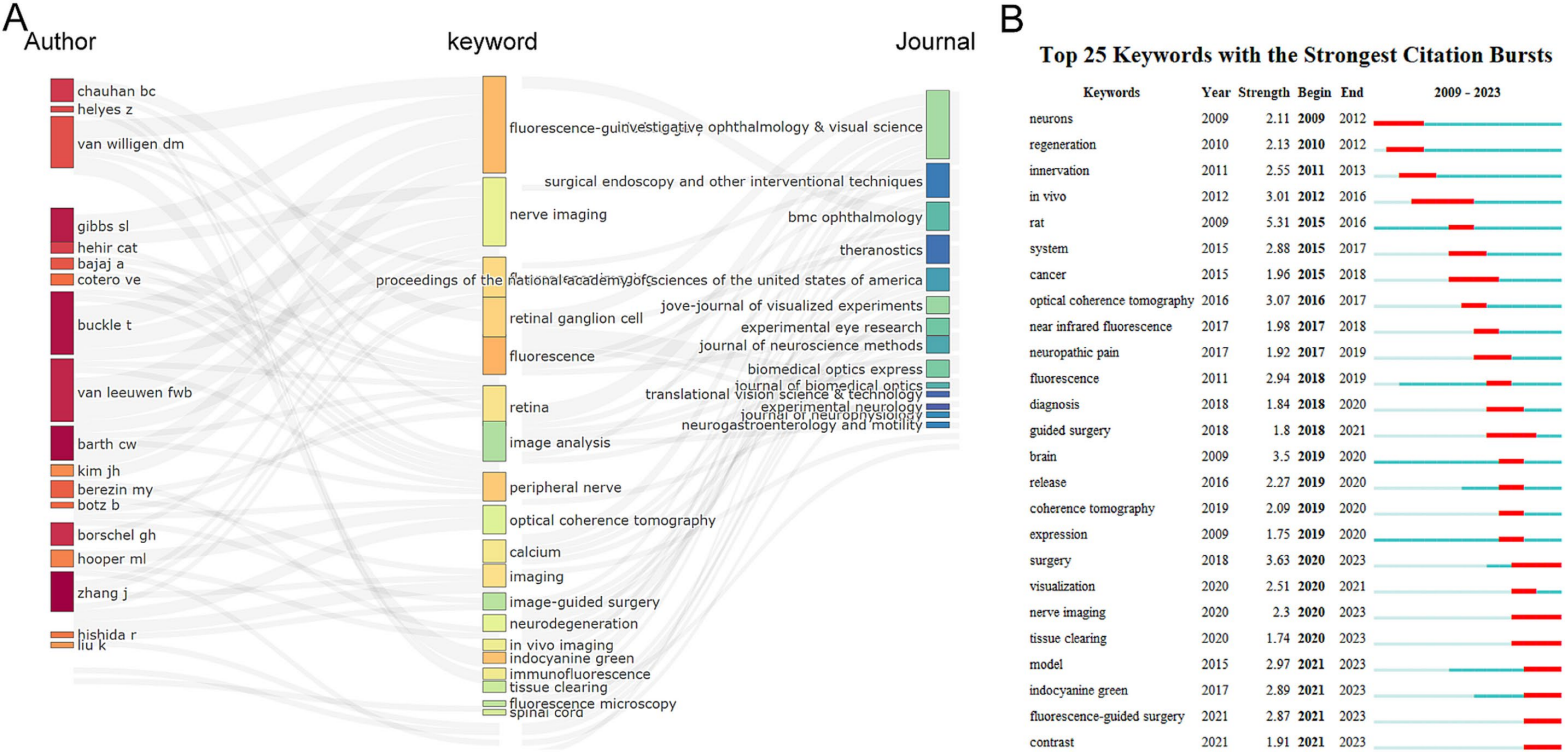
**(A)** Three-field plot of the keywords analysis by R software. Middle field: keywords; Left field: authors; Right field: journals. **(B)** Top 25 keywords with the strongest citation bursts of publications based on CiteSpace.

Figure 8A, generated using R package “bibliometrix,” displays a Three-Field Plot where authors, keywords, and journals are linked. Through this three-field plot, one can observe the connections between the main elements, which are directly represented by the strength of the connecting links. The most frequently used keywords are “fluorescence-guided surgery,” “nerve imaging,” and “fluorescence,” which align with the keywords presented in Figure 7. In particular, the top three authors based on publication volume, Van Willigen DM, Buckle T, and Van Leeuwen FWB, all have the strongest connections to the keyword “fluorescence-guided surgery,” as shown in this figure. Meanwhile, we can also find that keywords “fluorescence” and “image analysis” establish the strongest associations with the published journals. Therefore, such visualization suggests that fluorescence-guided surgery and image analysis can be considered hot topics in the current field and are of great significance for further research in this area.

We conducted an investigation of keyword bursts using the CiteSpace algorithm, and the results displayed the top 25 keywords with the strongest citation bursts (Figure 8B). The keyword with the strongest burst was “rat” (strength = 5.31), followed by “surgery” (strength = 3.63) and “brain” (strength = 3.5). The keyword “in-vivo” had the longest burst duration, spanning 4 years from 2012 to 2016. The majority of the keywords were published prior to 2020, whereas there are comparatively novel keywords that have emerged subsequent to 2021 include “model,” “indocyanine green,” “fluorescence-guided surgery,” and “contrast.” Interestingly, the keywords “surgery,” “nerve imaging,” and “tissue clearing” had the highest burst frequency from 2020 to 2023, indicating that these topics are likely potential research hotspots in the future.

## 4 Discussion

In the past decade, OMI techniques has garnered significant global research interest and has been proven to be an efficient technique for precise identification and localization of target tissues in surgical procedures (24, 25). Compared to conventional imaging techniques, OMI techniques offer notable advantages in imaging depth and clarity without causing harm to human tissues. This technology can be utilized in clinical diagnosis, analyzing biological phenomena, monitoring molecular content, observing the distribution of target tissues, and visualizing different biological structures. In this study, we conducted a seminal bibliometric analysis of publications related to OMI techniques and its application in nerve imaging, based on literature from 2009 to 2023, using CiteSpace and VOSviewer. Our analysis highlights the latest research trends and potential future hotspots in this rapidly advancing field.

### 4.1 Trends of related research publications in this field

Our study revealed that the number of publications in nerve imaging guided by OMI techniques has shown a linear growth trend over the past decade. From January 1, 2009, to December 31, 2023, a total of 35 countries worldwide contributed to the literature in this field. Among them, the USA published 185 papers, accounting for 44.69% of the total publications, making it the country with the highest contribution in this field. Among the top-ranking countries, the Netherlands, ranking sixth in terms of publication quantity, has shown significant progress in the total citation field. Although the Britain ranked sixth in publication quantity, it had the highest average citation count, indicating potentially higher output quality or impact. The USA had an average citation count of 26.10, second only to Britain, suggesting that the research output from the USA not only demonstrates high productivity but also considerable influence, indicating its leading position in this field. Despite being the second-highest country in terms of overall publications, China demonstrated comparatively lower performance in average citation counts, indicating that the research quality in this field from China may not be as high, possibly making it challenging for China to surpass the USA in the coming decades. Further investigation is warranted to examine the paradoxical relationship between the quantity and quality of publications originating from China. In terms of research institutions, Shanghai Jiao Tong University made the most contributions in this field with 11 papers. It is worth mentioning that all of the top 10 research institutions hail from the top five countries. This observation highlights the significant influence exerted by top-tier research institutions on enhancing a country’s academic research ranking. Oregon Health and Science University had the highest average citation count (32.75), followed by the University of Toronto (29.71) and the University of Miami (25.75). Two of these institutions are from the USA, reaffirming the leading position of the USA in this research field. Interestingly, while Shanghai Jiao Tong University had the highest total publication count, its average citation count (9.27) was not outstanding compared to other institutions. The USA and China were the two countries with the most frequent collaborations. Additionally, Shanghai Jiao Tong University, Stanford University, and Oregon Health and Science University were the top three institutions in terms of collaborations with other institutions, all from the USA and China. Hence, this combined evidence strongly indicates that exploring collaborations can significantly influence researchers in producing top-notch papers within this domain.

The analysis of major journals in this field reveals that Investigative Ophthalmology and Visual Science, Jove-Journal of Visualized Experiments, and PLoS One are the top three contributing journals. It is interesting to observe that these journals have relatively low impact factors. This may suggest a trend where authors are inclined to use Open Access, allowing their publications to be quickly and widely accessed by readers. Furthermore, comparing the impact factor distribution of the top 10 journals with the number of publications, it is notable that top-tier academic journals such as Nature and Science also appear in the list. This reflects researchers’ emphasis on literature from traditional high-impact journals during the research process. Considering the nature of OMI techniques, which involves physics, chemistry, and biomaterials, it is observed that some of the top-ranked journals are associated with not only medical but also physics, chemistry, and biomaterials disciplines. Our analysis of the citation count for these journals aligns with the aforementioned findings. Additionally, a dual-map overlay of journals further supports this observation.

In terms of authors, the most prolific contributors are from the USA. It’s worth noting that the USA Department of Health Human Services provides the most funding, indicating the significant role played by the USA in this field. Among the eight authors with more than five publications, five of them also have their total citation counts ranked within the top 10. This may be attributed to their early entry into this field, leading to greater attention to their latest research findings.

Furthermore, Figure 5C reveals that the analysis of collaboration demonstrates a scattered distribution of collaborations among authors originating from diverse nations. This signifies a dearth of scholarly exchanges and connections between researchers from varying countries, which undeniably impedes the progress of the field. Hence, it becomes imperative for authors hailing from different nations and institutions to intensify their collaborations, thereby fostering collective advancement in research within this domain.

The most cited article in this field is a study titled “Rapid transport within cerebral perivascular spaces underlies widespread tracer distribution in the brain after intranasal administration” published in 2015 (26), followed by is a research titled “*In vivo* imaging reveals a phase-specific role of STAT3 during central and peripheral nervous system axon regeneration” published in 2011 (27).

The three most co-cited articles are focused on the topics of nerve imaging, fluorescence-guided surgery, and preclinical studies of fluorescent probes (28–30). Through co-occurrence analysis of the included references, these popular topics have been validated. These studies were categorized into 11 clusters, mainly related to “image-guided surgery” and “fluorescence-guided surgery,” indicating that these directions are the hotspots in this research field.

### 4.2 Research hotspots and frontiers

The analysis of keyword co-occurrence and bursts reveals the current development trends and key areas of focus in this field. As shown in Figure 8B, “rat” is the most frequently cited keyword, representing its initial state in research within this field. In our investigation, we establish the co-occurring keyword network using the titles and abstracts of all incorporated publications. The clustering of these four significant research directions is demonstrated in Figure 7A, segregating them into four distinct groups: Fluorescence probes (red), Image-guided surgery (blue), Nerve tissue engineering (green), and Axons (yellow). These findings indicate both the alignment of this field with current research hotspots and potential future research directions, as described below.

#### 4.2.1 Fluorescence probes

The results of the keyword contribution analysis indicate that relevant terms for Fluorescence probes include “fluorescence,” “mechanisms,” and “microscopy.” Fluorescence probes are molecules or substances that can emit fluorescence signals. They can quantitatively or qualitatively analyze target molecules by binding or reacting with specific target molecules (31, 32). In the past decade, fluorescence probes have been widely used in disease qualitative analysis and quantitative sensing due to their high sensitivity and spatial resolution (33, 34). Fluorescence microscopy is a powerful tool for visualizing biomolecules and cellular structures at the nanoscale (35), which expands the application of fluorescence probes in studying various cellular processes at the molecular level (36). Specifically, fluorescence probes target organelles for high-precision detection of cellular metabolites and transient chemical messengers, making them valuable tools for studying biochemical pathways. Fluorescence microscopy enables dynamic measurements of neuronal function with extremely high spatial and temporal resolutions, successfully revealing the molecular mechanisms underlying neurophysiological processes in neuronal cell cultures and animal models (37). This contributes to researchers observing and recording the activities and mechanisms of neurons using fluorescence probes, thereby gaining in-depth understanding of the functionality and disease mechanisms of the nervous system. It holds significant application value in fields such as neuroscience research, neuropharmacology, nerve tissue imaging, and brain imaging.

#### 4.2.2 Image-guided surgery

Image-guided surgery is a method that utilizes medical imaging techniques to guide and assist in surgical procedures. It combines advanced imaging technology with computer navigation systems to provide real-time anatomical information and guidance for surgeons, aiming to achieve more accurate, safe, and effective surgical operations. In order to guide surgical procedures effectively, a range of imaging techniques are utilized like fluorescence imaging, ultrasound, CT, MRI, nuclear medicine imaging, and nanotechnology imaging (38). Each imaging modality has its sensitivity, resolution, quantitative capabilities, as well as a range of advantages and inherent limitations. The results of keyword co-occurrence analysis indicate hotspots related to biomaterials, including “in-vivo,” “identification,” and “fluorescence-guided surgery.” Fluorescence-guided surgery has achieved significant success in the precise identification and resection of tumors. Intraoperative image-guided surgery often favors the utilization of near-infrared fluorescence imaging within the range of 700–900 nm wavelengths. This choice stems from its superior capability to penetrate tissue, combined with minimal scattering and autogenous absorption when compared to visible light spectroscopy (39–41).

The ICG is currently the only near-infrared fluorescence dye approved by the Food and Drug Administration and the European Medicines Agency for a few surgical indications (42). Gragnaniello et al. (43) identified and preserved the facial nerve and semicircular canals in fresh human head specimens via arterial injection of indocyanine green under near-infrared fluorescence imaging. In 2014, based on the difference between cortical bone and the facial nerve, Chen et al. (44) diluted and intravenously injected indocyanine green into the facial nerve canal’s accompanying vessels after ossification during mastoidectomy, visualizing the nerve. This helps surgeons locate it precisely and reduces injury complications. Weng et al. (45) found thoracic sympathetic ganglia could be clearly shown in near-infrared mode during thoracoscopic indocyanine green imaging, the first such report with no patient adverse reactions. Later, the team explored optimal injection time and dose (46). Jin et al. (47) visualized certain nerve plexuses during radical colectomy. Kanno et al. (48) used it in a deep endometriosis operation, highlighting relevant nerves with no postoperative nerve injury. From 2020 to 2021 He et al. (49) evaluated the feasibility and safety of using near-infrared fluorescence imaging and indocyanine green to identify pelvic nerves in radical hysterectomy for cervical cancer. Yang et al. (50) and Hao et al. (51) achieved precise identification and resection of bladder cancer *in vivo* and *ex vivo* tissues through ICG coupled with tumor targets.

The successful application of fluorescence imaging technology has accelerated the development of image-guided surgery, gradually bringing surgical procedures into the realm of precision surgery. Due to the structural differences in nerve tissue, ICG faces difficulties in targeted binding to nerve tissue. Therefore, some researchers suggest that the ideal nerve fluorescence probe would be a conjugate of ICG with nerve-specific targets (52).

#### 4.2.3 Nerve tissue engineering

Nerve tissue engineering involves the use of materials, biology, and engineering principles to study and design biomaterials and biomimetic models that can be used for nerve imaging. This multidisciplinary approach has gained significant attention in the field of nerve imaging. It is particularly important for the precise identification of complex nerve targets, such as neuronal cell labeling, microstructural markers, and fluorescence probe characterization (53, 54). For example, Ma’s team seeded adipose-derived stem cells labeled with fluorescent dye PKH26 onto decellularized nerve scaffolds, achieving neuronal cell tracking. This method allows real-time monitoring of the nerve tissue repair process (55). Gibbs et al. constructed a near-infrared nerve-specific fluorescence probe library by chemically modifying and modifying existing fluorescent dyes. This provides a novel fluorescence probe for intraoperative real-time identification of nerve tissue. One of the probes, LGW08-35, successfully underwent toxicology, pharmacokinetics, and pharmacodynamics testing in animal experiments, demonstrating its potential for clinical translation (56, 57).

#### 4.2.4 Axons

The main function of axons is to transmit nerve impulses, but they also participate in various other physiological and pathological processes, such as axonal transport, axon regeneration, and nerve repair. Axonal transport (AT) is a fundamental cellular process of neurons, playing a crucial role in the development and preservation of neuronal structures and connections (58). The development of new agents and histological methods has always been driven by specific needs. One such need is the development of agents that can be administered to specific brain regions *in vivo*, allowing for the subsequent visualization of neuronal connectivity through the AT of the agent. Fluorescence imaging of cranial nerves or peripheral nerves can be achieved using fluorescent agents such as Fast Blue, Fluorogold, NeuroTrace, and Dio/FastDio (59–63), which rely on anterograde and retrograde AT. Additionally, AT can be used to track the sites of action of neurotropic viruses (such as rabies virus, herpes simplex virus, adenovirus) (64, 65). By fluorescently imaging the axons, the extent of brain or nerve tissue lesions can be detected. Moreover, this provides guidance for the synthesis of nerve-specific fluorescence probes. Axon regeneration, as a significant step in nerve repair, can be dynamically monitored in real-time through fluorescent imaging of axons. This enables us to assess the progress of nerve tissue repair and provide patients with appropriate rehabilitation plans.

### 4.3 Research limitations and prospects

Our research has a number of limitations. Firstly, the collection of all literature was conducted exclusively from WoSCC, thereby potentially introducing selection bias due to reliance on a single database search. Secondly, we solely extracted studies in English, which may have caused us to overlook a substantial amount of relevant research published in other languages, particularly considering the significant contribution of China in this field. Additionally, in instances of disagreement during the data selection process, the final determination was made exclusively by seasoned corresponding author. Given the continuous influx of new research, there is a possibility that citation data for recently influential publications might have been disregarded, consequently resulting in some predictive bias when examining trends and keywords based on the included publications over time.

The OMI technology in the application of neural tissue tracing imaging marks a significant advancement in the field of neuronal science. By utilizing fluorescently labeled molecular probes, scientists are able to visually observe the structure and function of neurons, as well as the complex connections of neural networks, at the cellular and molecular levels. This not only deepens our understanding of the development, function, and mechanisms of neurological diseases but also shows great potential in drug development, research on neurodegenerative diseases, and navigation in neurosurgical operations. The development of OMI technology, especially the progress in near-infrared fluorescence imaging, provides deeper tissue penetration and higher spatial resolution imaging with lower tissue scattering and autofluorescence. This technological advancement has not only promoted the understanding of the fundamental mechanisms of the nervous system but also offered new possibilities for clinical diagnosis and treatment. With the continuous development of new fluorescent probes and imaging systems, OMI will play an increasingly important role in neuronal science research and clinical applications, potentially bringing revolutionary changes to the diagnosis and treatment of neurological diseases. In terms of neuronal activity monitoring, scientists can detect changes in neural activity in real-time using fluorescent dyes such as calcium indicators, providing dynamic data for the study of neural information processing (66). In neuronal development research, the technology can track the differentiation and migration of neural precursor cells and observe the integration of new neurons, offering vital information for understanding the development of the nervous system (67). In research on neurodegenerative diseases, OMI monitors pathological changes such as amyloid plaques and neurofibrillary tangles, contributing to the elucidation of disease mechanisms (68–70). In the field of drug development and screening, the technology assesses the impact of drugs on the structure and function of neurons, accelerating drug development and screening, and providing new strategies for the treatment of neurological diseases (71). In neurosurgery, OMI assists surgeons in identifying and protecting key neural structures, reducing neural damage during surgery, and enhancing surgical safety (72). In the future, with the continuous advancement of imaging technology and fluorescent probes, OMI is expected to play a more critical role in neuroscientific research and clinical applications, bringing new breakthroughs to the diagnosis and treatment of neurological diseases.

## 5 Conclusion

This investigation represents the primary exhaustive bibliometric examination of worldwide research patterns in nerve imaging guided by OMI techniques. In this study, a systematic compilation of global publication patterns is conducted, which can assist researchers in determining influential authors, institutions, and journals. Moreover, keyword cluster analysis serves as a guidance tool for researchers to explore novel research avenues. The focal areas of investigation encompass “surgery guided by fluorescence,” “spinal cord,” “intraoperative imaging,” and “pyridinic-n-rich-carbon dots.” Attaining an understanding of ongoing research in this rapidly expanding field will enable researchers to augment their knowledge base and push the boundaries of the discipline.

## Data Availability

The original contributions presented in the study are included in the article/Supplementary material, further inquiries can be directed to the corresponding authors.
