# Supplementary material for: Evolution in optical molecular imaging techniques guided nerve imaging from 2009 to 2023: a bibliometric and visualization analysis
Source: Front Neurol. 2025 Jan 22;15:1474353. doi: 10.3389/fneur.2024.1474353 (PMC11794114; doi:10.3389/fneur.2024.1474353)
Supplement: Supplementary file 1 [file Data_Sheet_1.docx]

Table S1. Top 10 countries that contributed publications

| Rank | Countries | Record Count | Percentage (%, N/414) | Total Citations | Average Citation |
| --- | --- | --- | --- | --- | --- |
| 1 | USA | 185 | 44.69 | 4,828 | 26.10 |
| 2 | CHINA | 93 | 22.46 | 1,590 | 17.10 |
| 3 | GERMANY | 42 | 10.15 | 857 | 20.40 |
| 4 | JAPAN | 38 | 9.18 | 589 | 15.50 |
| 5 | CANADA | 26 | 6.28 | 555 | 21.35 |
| 6 | BRITAIN | 19 | 4.59 | 764 | 40.21 |
| 7 | SOUTH KOREA | 17 | 4.11 | 184 | 10.82 |
| 8 | AUSTRALIA | 13 | 3.14 | 228 | 17.54 |
| 9 | FRANCE | 12 | 2.90 | 150 | 20.83 |
| 10 | ITALY | 10 | 2.42 | 212 | 21.20 |

Table S2. Top 10 institutions that contributed publications

| Rank | Institution | Country | Record Counts | Percentage (%, N/414) | Total Citations | Average Citation |
| --- | --- | --- | --- | --- | --- | --- |
| 1 | Shanghai Jiao Tong University | CHINA | 11 | 2.66% | 102 | 9.27 |
| 2 | Stanford University | USA | 9 | 2.17% | 116 | 12.89 |
| 3 | Oregon Health and Science University | USA | 8 | 1.93% | 262 | 32.75 |
| 4 | University Miami | USA | 8 | 1.93% | 206 | 25.75 |
| 5 | Fudan University | CHINA | 7 | 1.69% | 191 | 27.29 |
| 6 | University of California, San Diego | USA | 7 | 1.69% | 104 | 14.86 |
| 7 | University Toronto | CANADA | 7 | 1.69% | 208 | 29.71 |
| 8 | Washington University | USA | 7 | 1.69% | 112 | 16.00 |
| 9 | Chinese Academy of Sciences | CHINA | 6 | 1.45% | 154 | 25.67 |
| 10 | Dalhousie University | CANADA | 6 | 1.45% | 45 | 7.50 |

Table S3. Top 10 authors with the most publications

| Rank | Author | Country | Record Counts |
| --- | --- | --- | --- |
| 1 | Zhang J | CHINA | 7 |
| 2 | Barth C | USA | 6 |
| 3 | Buckle T | NETHERLANDS | 6 |
| 4 | Gibbs SL | USA | 6 |
| 5 | Van Leeuwen FWB | NETHERLANDS | 6 |
| 6 | Borschel G | CANADA | 5 |
| 7 | Chauhan BC | CANADA | 5 |
| 8 | Hehir CAT | USA | 5 |
| 9 | Helyes Z | HUNGARY | 5 |
| 10 | Van Willigen DM | NETHERLANDS | 5 |
